# Human Umbilical Cord Mesenchymal Stem Cell‐Derived Exosomes Preserve Cartilage Homeostasis and Restore Joint Function in Experimental Osteoarthritis

**DOI:** 10.1002/kjm2.70312

**Published:** 2026-11-05

**Authors:** Sung‐Yen Lin, Yi‐Shan Lin, Shu‐Chun Chuang, Yi‐Wen Kuo, Cheng‐Jung Ho, Kai‐Hsuan Wang, Wei‐Cheng Tsai, Zhi‐Xiang Li, Wan‐Jung Lin, Yi‐Ju Hsieh, Chung‐Hwan Chen, Tzou‐Yien Lin

**Affiliations:** ^1^ Orthopaedic Research Center Kaohsiung Medical University Kaohsiung Taiwan; ^2^ Regenerative Medicine and Cell Therapy Research Center Kaohsiung Medical University Kaohsiung Taiwan; ^3^ Department of Orthopaedics, Kaohsiung Medical University Hospital Kaohsiung Medical University Kaohsiung Taiwan; ^4^ Department of Orthopedics Kaohsiung Medical University Gangshan Hospital Kaohsiung Taiwan; ^5^ Department of Orthopedics, School of Post‐Baccalaureate Medicine, College of Medicine Kaohsiung Medical University Kaohsiung Taiwan; ^6^ Department of Orthopaedic Surgery University of Virginia Charlottesville Virginia USA; ^7^ ExoOne Bio Co., Ltd. Taipei Taiwan; ^8^ School of Medicine, College of Medicine Kaohsiung Medical University Kaohsiung Taiwan; ^9^ Ph.D. Program in Biomedical Engineering, College of Medicine Kaohsiung Medical University Kaohsiung Taiwan; ^10^ Department of Materials Engineering, College of Engineering National Pingtung University of Science and Technology Pingtung Taiwan; ^11^ Department of Pediatrics, Division of Infectious Diseases Chang Gung Memorial Hospital Taoyuan Taiwan; ^12^ College of Public Health Taipei Medical University Taipei Taiwan

**Keywords:** exosome, human umbilical cord mesenchymal stem cells, mesenchymal stem cell, osteoarthritis

## Abstract

Mesenchymal stem cells (MSCs) have shown therapeutic potential for osteoarthritis (OA), but their clinical application is limited by the need for extensive ex vivo expansion. Human umbilical cord MSC‐derived exosomes (hUCMSC‐Exos) have emerged as a promising cell‐free alternative because of their cartilage‐protective and immunomodulatory properties. hUCMSC‐Exos were characterized by nanoparticle tracking analysis and evaluated in an interleukin‐1β (IL‐1β)‐stimulated human chondrocyte model and an anterior cruciate ligament transection (ACLT)‐induced rat OA model. In vitro, hUCMSC‐Exos suppressed inflammatory and catabolic responses while enhancing extracellular matrix synthesis, as evidenced by reduced IL‐1β, MMP13, and Type X collagen expression and increased TIMP1, aggrecan, and Type II collagen expression. In vivo, repeated intra‐articular administration of hUCMSC‐Exos attenuated cartilage degeneration, resulting in improved gross joint morphology, reduced histopathological damage, increased glycosaminoglycan deposition and Type II collagen expression, and decreased MMP13 expression. These structural improvements were accompanied by enhanced functional recovery, as demonstrated by improved hind limb weight‐bearing and treadmill running endurance. These findings suggest that hUCMSC‐Exos alleviate cartilage degeneration and improve joint function, supporting their potential as a promising cell‐free therapeutic approach for OA.

## 1 Introduction

Osteoarthritis (OA) is the most common degenerative joint disease and a leading cause of chronic pain and disability worldwide. According to the Global Burden of Disease Study 2021, more than 595 million people are affected by OA globally, and the burden continues to increase because of population aging and the rising prevalence of obesity [1, 2]. Knee OA is the major contributor to OA‐related disability and substantially impairs mobility, physical function, and quality of life. Although current pharmacological treatments provide symptomatic relief, none effectively halt or reverse progressive cartilage degeneration, and many patients eventually require total knee arthroplasty. Consequently, developing disease‐modifying therapies capable of preserving joint structure and function remains a major unmet clinical need.

Mesenchymal stem cells (MSCs) have attracted considerable attention as regenerative therapies because of their immunomodulatory, anti‐inflammatory, and trophic properties. Increasing evidence suggests that the therapeutic benefits of MSCs are mediated predominantly through paracrine signaling rather than durable engraftment [3]. Exosomes represent an important component of this paracrine activity and can transfer proteins, lipids, mRNAs, and microRNAs to recipient cells [4]. Compared with conventional MSC transplantation, MSC‐derived exosomes provide a cell‐free therapeutic strategy that may retain key paracrine functions of MSCs while avoiding concerns related to viable‐cell engraftment, uncontrolled proliferation, or differentiation. Exosomes also offer practical advantages including lower immunogenicity, improved storage stability, easier product characterization and quality control, and greater feasibility for standardized large‐scale manufacturing [5, 6].

Human umbilical cord‐derived MSCs (hUCMSCs) represent an especially promising source of therapeutic exosomes because they can be obtained non‐invasively, exhibit high proliferative capacity, and possess favorable immunomodulatory properties [7]. Previous studies have demonstrated that hUCMSC‐derived exosomes (hUCMSC‐Exos) suppress inflammatory responses, inhibit chondrocyte apoptosis and oxidative stress, enhance extracellular matrix synthesis, and reduce cartilage catabolism through downregulation of matrix‐degrading enzymes such as MMP13 and ADAMTS5 [8, 9]. In surgically induced OA models, intra‐articular injection of hUCMSC‐Exos has been shown to preserve cartilage integrity, maintain extracellular matrix composition, and improve histological outcomes [8, 9]. However, therapeutic efficacy has been evaluated predominantly using molecular and histological endpoints. Whether these structural improvements translate into meaningful restoration of joint function remains largely unknown, despite functional recovery representing the ultimate therapeutic goal for patients with OA.

Therefore, the present study aimed to investigate whether hUCMSC‐Exos preserve cartilage homeostasis while simultaneously improving joint function in experimental OA. We hypothesized that suppression of cartilage catabolism together with enhancement of extracellular matrix restoration would translate into functional recovery. To test this hypothesis, we evaluated the therapeutic effects of hUCMSC‐Exos using both in vitro and in vivo OA models, with particular emphasis on whether molecular and structural cartilage preservation translates into functional improvement.

## 2 Materials and Methods

### 2.1 Isolation and Characterization of hUCMSC‐Exos

hUCMSCs were obtained from Taiwan Bio Therapeutics Co. Ltd. (Hsinchu, Taiwan). According to the manufacturer, hUCMSCs were isolated from Wharton's jelly of donated umbilical cords after written informed consent and approval by the Taipei Medical University Joint Institutional Review Board (TMU‐Joint IRB No. N202110023). Cell identity was authenticated by short tandem repeat profiling, confirmed to be mycoplasma‐free, and characterized by standard MSC surface marker expression.

For exosome production, hUCMSCs were cultured in α‐minimum essential medium supplemented with human platelet lysate under standard culture conditions (37°C, 5% CO_2_). Conditioned medium was collected at 80%–90% confluence, filtered through a 0.22‐μm membrane, and exosomes were isolated by tangential flow filtration using a 100‐kDa molecular weight cut‐off cassette (Sartorius, Göttingen, Germany). Purified hUCMSC‐Exos were stored at −80°C until use. Protein concentration was determined using a bicinchoninic acid assay, while particle size distribution and concentration were analyzed by nanoparticle tracking analysis (NTA) [10]. For morphological characterization, hUCMSC‐Exos were examined by transmission electron microscopy (TEM). Briefly, the exosome suspension was applied to a copper grid and air‐dried. Samples were fixed with 3% glutaraldehyde for 2 h, washed with phosphate‐buffered saline, and negatively stained with 2% uranyl acetate for 30 s. The vesicles were examined using a JEM‐1230 transmission electron microscope (JEOL, Tokyo, Japan), and images were captured using a CCD camera (SC1000W, Gatan). The protein‐marker profile of hUCMSC‐Exos was further characterized by Western blotting. hUCMSCs and isolated exosomes were lysed in radioimmunoprecipitation assay (RIPA) buffer supplemented with protease inhibitors. Protein lysates were separated by sodium dodecyl sulfate‐polyacrylamide gel electrophoresis (SDS‐PAGE) and transferred onto polyvinylidene difluoride (PVDF) membranes. After blocking, membranes were incubated overnight at 4°C with primary antibodies against CD9 (Cell Signaling, #13174), CD63 (Abcam, ab134045), CD81 (GeneTex, GTX101766), CD73 (Proteintech, 12231‐1‐AP), HSP70 (Cell Signaling, #4872), TSG101 (Cell Signaling, #72312), and calnexin (Cell Signaling, #2679), all at 1:1000 dilution, followed by horseradish peroxidase (HRP)‐conjugated secondary antibodies (Proteintech Group, USA) and enhanced chemiluminescence (ECL) detection.

### 2.2 Cell Culture and In Vitro Experiments

Human articular chondrocytes were stimulated with interleukin‐1β (IL‐1β, 10 ng/mL) in the presence or absence of hUCMSC‐Exos (10^8^ or 10^9^ particles/mL). Cell viability was evaluated after 5 days using the CellTiter 96 AQueous One Solution Cell Proliferation Assay (Promega, Madison, WI, USA) according to the manufacturer's instructions [11]. Total RNA was extracted using TRIzol reagent (Invitrogen, Carlsbad, CA, USA), reverse‐transcribed into complementary DNA, and analyzed by quantitative real‐time PCR using SYBR Green chemistry. Relative expression of IL1B, MMP13, COL10A1, TIMP1, ACAN, and COL2A1 was normalized to glyceraldehyde‐3‐phosphate dehydrogenase (GAPDH) using the 2^−ΔΔCt^ method. Primer sequences are provided in Supporting Information. Extracellular matrix synthesis was assessed by Alcian blue staining and quantified using the dimethylmethylene blue (DMMB) assay. Sulfated glycosaminoglycan (sGAG) content was normalized to DNA content to account for differences in cell number.

### 2.3 Animal Model and Treatment

All animal procedures were approved by the Institutional Animal Care and Use Committee of Kaohsiung Medical University (Approval No. 111054). Twenty‐four male Sprague–Dawley rats (11 weeks old) were randomly assigned to four groups (*n* = 6/group): Sham, OA, OA + hUCMSC‐Exos (10^9^ particles), and OA + hUCMSC‐Exos (10^10^ particles). OA was induced by anterior cruciate ligament transection (ACLT) of the right knee. At 3 weeks after surgery, rats received intra‐articular injections of vehicle or hUCMSC‐Exos (10^9^ or 10^10^ particles in 40 μL) every 3 days for 5 weeks while maintaining unrestricted cage activity.

### 2.4 Functional Assessment

Pain‐related functional recovery was evaluated using hind limb weight‐bearing distribution and treadmill endurance tests. Weight‐bearing distribution, an indicator of joint pain behavior, was measured using a dual‐channel weight averager (Singa Technology, Taipei, Taiwan). Each hind paw was placed on a separate force plate within a Plexiglas chamber, and force was recorded over 5 s. Measurements were obtained in triplicate at baseline (pre‐surgery) and weekly after ACLT. Weight‐bearing asymmetry was calculated as the difference between the operated (right) and contralateral (left) hind limbs [12]. Rats were acclimated to treadmill running (Columbus Instruments, Columbus, OH, USA) for 1 week before surgery (15 min/day at 10–15 m/min). After treatment, treadmill endurance was assessed twice within 1 week, and the mean running time was used for analysis. During testing, rats ran at a constant speed of 40 m/min for up to 10 min. A mild, non‐injurious electrical stimulus (0.2 mA, 400 V, 1 Hz) was applied only when necessary to maintain running compliance [12, 13, 14].

### 2.5 Histological Analysis

Knee joint specimens were fixed in 10% neutral‐buffered formalin for 24 h, decalcified in 10% formic acid, paraffin‐embedded, and sectioned coronally at 5 μm thickness. Cartilage glycosaminoglycan (GAG) content was assessed by Safranin O/Fast Green staining (1% Safranin O with hematoxylin and 0.01% Fast Green; Sigma‐Aldrich, St. Louis, MO, USA). Quantitative analysis of cartilage GAG content was performed using Image‐Pro Plus 5.0 (Media Cybernetics, Rockville, MD, USA) by calculating the percentage of Safranin O‐positive cartilage area relative to the total cartilage area. OA severity was evaluated using the Osteoarthritis Research Society International (OARSI) histopathology scoring system [12].

### 2.6 Immunohistochemical Staining for Type II Collagen and MMP13

Paraffin sections were deparaffinized in xylene, rehydrated through graded ethanol, and treated with 3% hydrogen peroxide to block endogenous peroxidase activity. After blocking with fetal bovine serum for 1 h at room temperature, sections were incubated with primary antibodies against Type II collagen (ab34712, Abcam, Cambridge, UK) and MMP13 (ab39012, Abcam) at 37°C for 4 h. Immunoreactivity was visualized using the EXPOSE HRP‐DAB kit (Abcam) according to the manufacturer's protocol, followed by hematoxylin counterstaining. Positive staining was quantified using Image‐Pro Plus 5.0 and expressed as the percentage of positively stained cartilage area.

### 2.7 Statistical Analysis

Data are presented as the mean ± standard error of the mean (SEM). Statistical analyses were performed using SPSS version 22.0 (IBM Corp., Armonk, NY, USA). Differences among groups were analyzed using one‐way analysis of variance (ANOVA) followed by Scheffé's post hoc test. A two‐sided *p* value < 0.05 was considered statistically significant.

## 3 Results

### 3.1 Characterization of hUCMSC‐Exos

Multiple complementary approaches confirmed the characteristics of the isolated hUCMSC‐Exos. NTA demonstrated a homogeneous vesicle population with a unimodal size distribution and a mean particle diameter of 104.6 ± 1.0 nm (Figure 1A). TEM revealed membrane‐bound vesicular structures with the characteristic morphology and size range of small extracellular vesicles (Figure 1B). Western blot analysis further demonstrated the presence of CD9, CD63, CD81, TSG101, HSP70, and CD73, whereas calnexin was not detected in the exosome fraction (Figure 1C).

**FIGURE 1:**
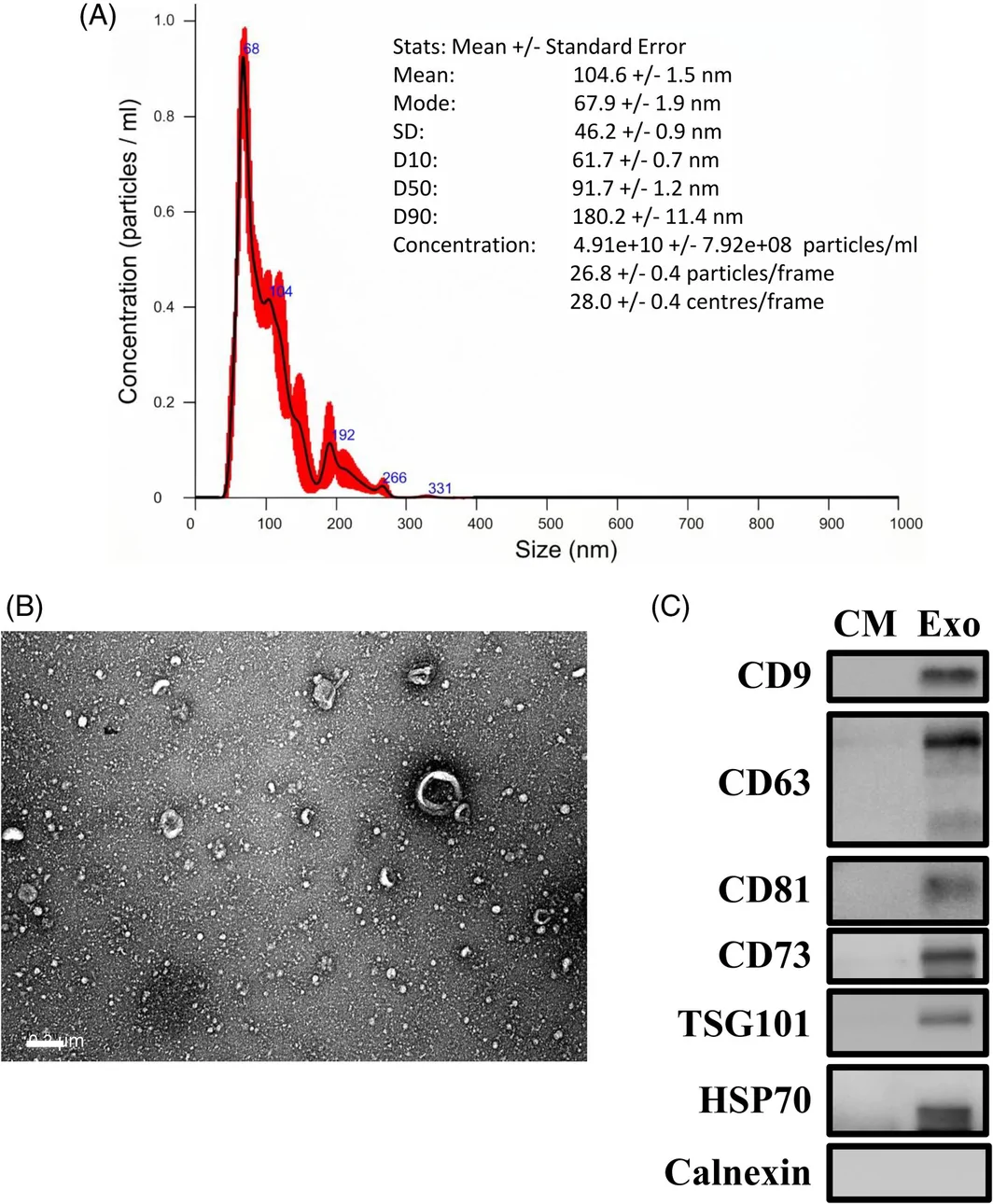
Characterization of human UCMSC‐derived exosomes by NTA, TEM, and Western blotting. (A) Nanoparticle tracking analysis (NTA) demonstrated a homogeneous size distribution with a mean particle diameter of 104.6 ± 1.0 nm. (B) Transmission electron microscopy (TEM) showed membrane‐bound vesicular structures characteristic of small extracellular vesicles. (C) Western blotting demonstrated CD9, CD63, CD81, TSG101, HSP70, and CD73, whereas calnexin was not detected in the exosome fraction.

### 3.2 hUCMSC‐Exos Preserve Chondrocyte Viability and Extracellular Matrix Homeostasis Under Inflammatory Conditions

To determine whether hUCMSC‐Exos protect chondrocytes from inflammatory injury, human articular chondrocytes were stimulated with IL‐1β in the presence or absence of exosomes. Exposure to IL‐1β significantly reduced chondrocyte viability, whereas hUCMSC‐Exos significantly improved cell viability at both 10^8^ and 10^9^ particles/mL compared with the IL‐1β‐treated group (both *p* < 0.01; Figure 2). Similarly, IL‐1β markedly reduced sGAG accumulation, whereas both hUCMSC‐Exo concentrations significantly restored sGAG production (Figure 3). The magnitude of these protective effects was generally comparable between the two concentrations.

**FIGURE 2:**
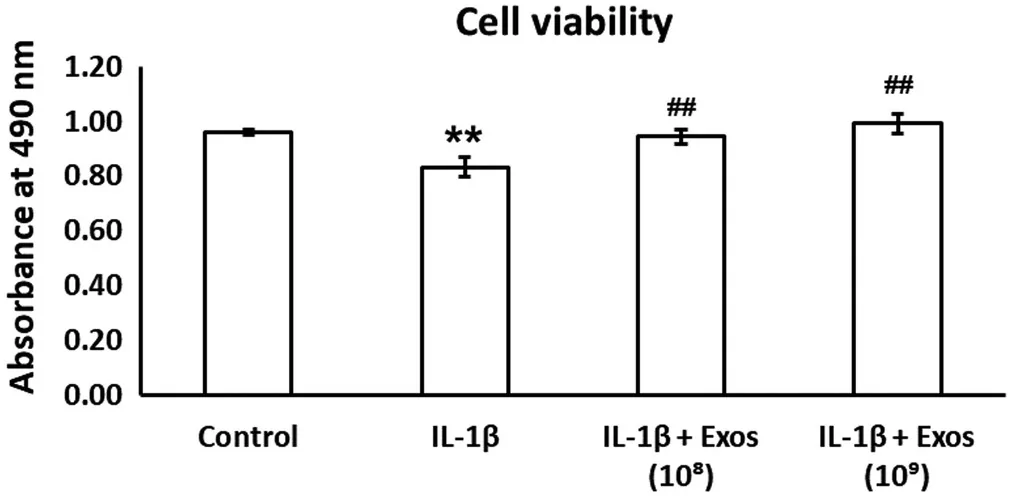
Human UCMSC‐derived exosomes preserve chondrocyte viability under IL‐1β stimulation. Human articular chondrocytes were stimulated with IL‐1β in the presence or absence of human UCMSC‐derived exosomes (10^8^ or 10^9^ particles/mL). IL‐1β significantly reduced chondrocyte viability, whereas both exosome concentrations significantly restored cell viability compared with IL‐1β treatment alone. Cell viability was assessed using the MTS assay. Statistical analysis was performed using one‐way ANOVA followed by Scheffé's post hoc test. *p < 0.05, **p < 0.01 versus Control; ^#^ *p* < 0.05, ^##^ *p* < 0.01 versus IL‐1β.

**FIGURE 3:**
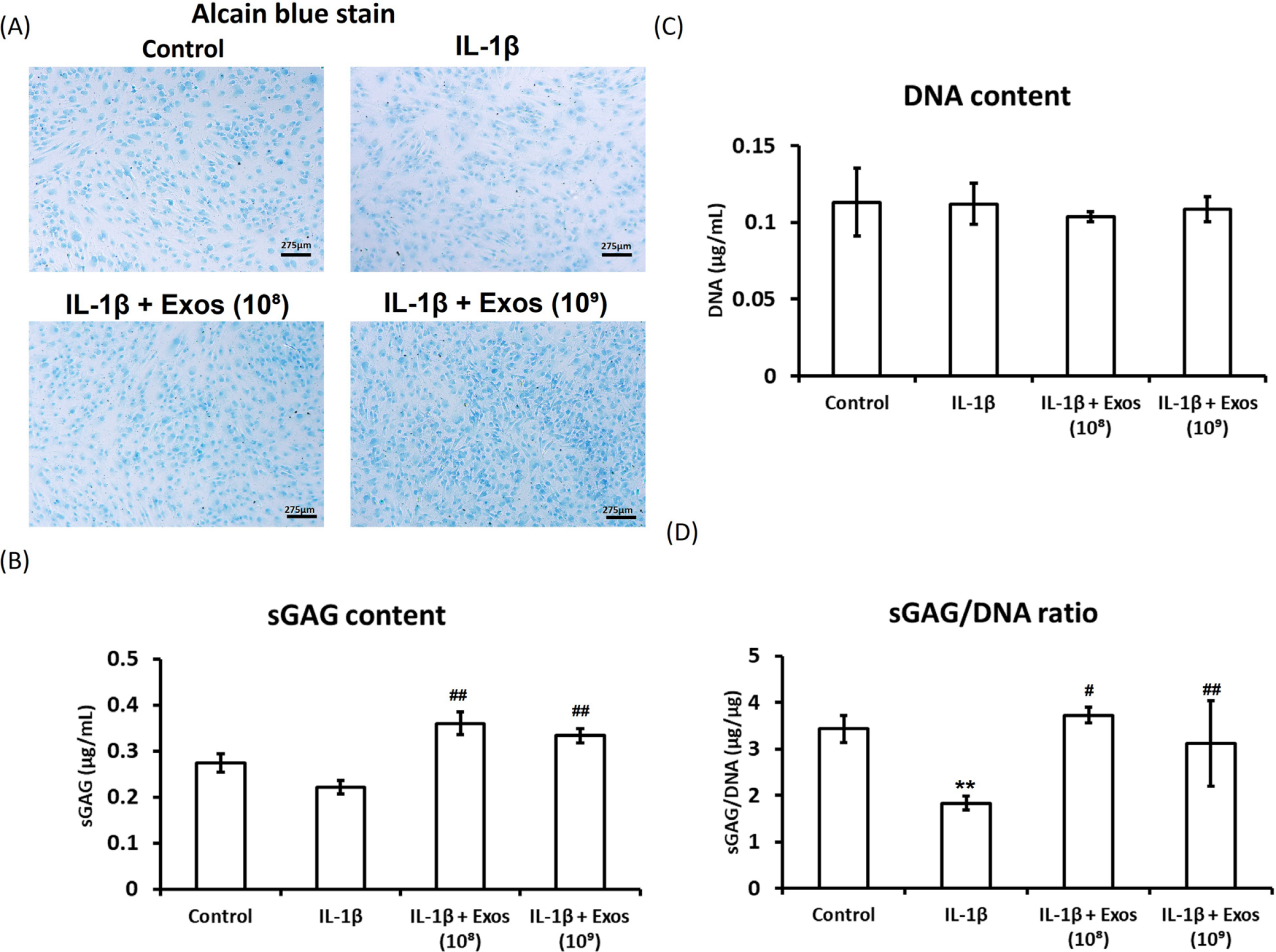
Human UCMSC‐derived exosomes restore extracellular matrix synthesis in IL‐1β‐stimulated chondrocytes. Human articular chondrocytes were stimulated with IL‐1β (10 ng/mL) and treated with hUCMSC‐derived exosomes (10^8^ or 10^9^ particles/mL). (A) Representative Alcian blue staining showing sulfated glycosaminoglycan (sGAG) deposition in human articular chondrocytes treated with IL‐1β (10 ng/mL) in the presence or absence of human UCMSC‐derived exosomes (10^8^ or 10^9^ particles/mL). (B) Quantification of sGAG content using the dimethylmethylene blue (DMMB) assay. (C) DNA content of cultured chondrocytes. (D) sGAG content normalized to DNA. Data are presented as the mean ± SEM. Statistical analysis was performed using one‐way ANOVA followed by Scheffé's post hoc test. *p < 0.05, **p < 0.01 versus Control; ^#^ *p* < 0.05, ^##^ *p* < 0.01 versus IL‐1β.

To further investigate the molecular effects of hUCMSC‐Exos, expression of OA‐related genes was analyzed by quantitative real‐time PCR. IL‐1β stimulation significantly increased the expression of the catabolic and hypertrophic markers IL‐1β, MMP13, and COL10A1, whereas hUCMSC‐Exos significantly suppressed their expression. In contrast, exosome treatment restored expression of the anabolic genes ACAN, COL2A1, and TIMP1, all of which were downregulated by IL‐1β stimulation (Figure 4). These findings indicate that hUCMSC‐Exos preserve cartilage homeostasis by suppressing inflammatory and catabolic responses while promoting extracellular matrix synthesis.

**FIGURE 4:**
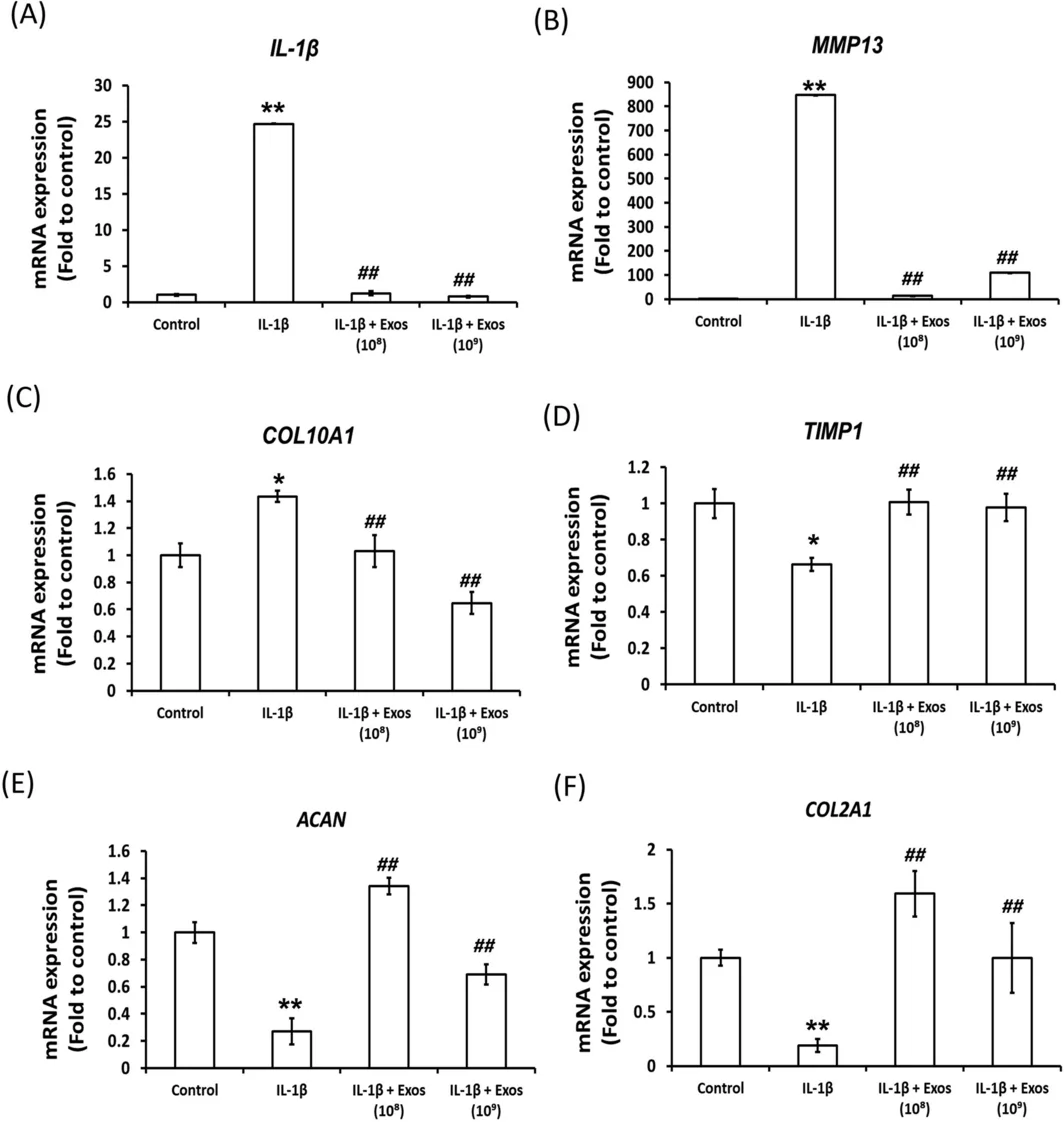
Human UCMSC‐derived exosomes regulate inflammatory and extracellular matrix‐related gene expression in IL‐1β‐stimulated chondrocytes. Human articular chondrocytes were stimulated with IL‐1β (10 ng/mL) in the absence or presence of human UCMSC‐derived exosomes (10^8^ or 10^9^ particles/mL). Relative mRNA expression of (A) *IL1B*, (B) *MMP13*, (C) *COL10A1*, (D) *TIMP1*, (E) *ACAN*, and (F) *COL2A1* were quantified by quantitative real‐time PCR and normalized to the control group. Data are presented as the mean ± SEM. Statistical analysis was performed using one‐way ANOVA followed by Scheffé's post hoc test. *p < 0.05, **p < 0.01 versus Control; ^#^ *p* < 0.05, ^##^ *p* < 0.01 versus IL‐1β.

### 3.3 hUCMSC‐Exos Improve Functional Recovery Following ACLT

Weight‐bearing asymmetry progressively increased following ACLT, indicating impaired joint function. Treatment with hUCMSC‐Exos significantly reduced weight‐bearing asymmetry compared with untreated OA animals throughout the treatment period (Figure 5A). Both hUCMSC‐Exo doses significantly improved treadmill endurance compared with untreated OA animals, with no significant difference between the 10^9^‐ and 10^10^‐particle groups (Figure 5B).

**FIGURE 5:**
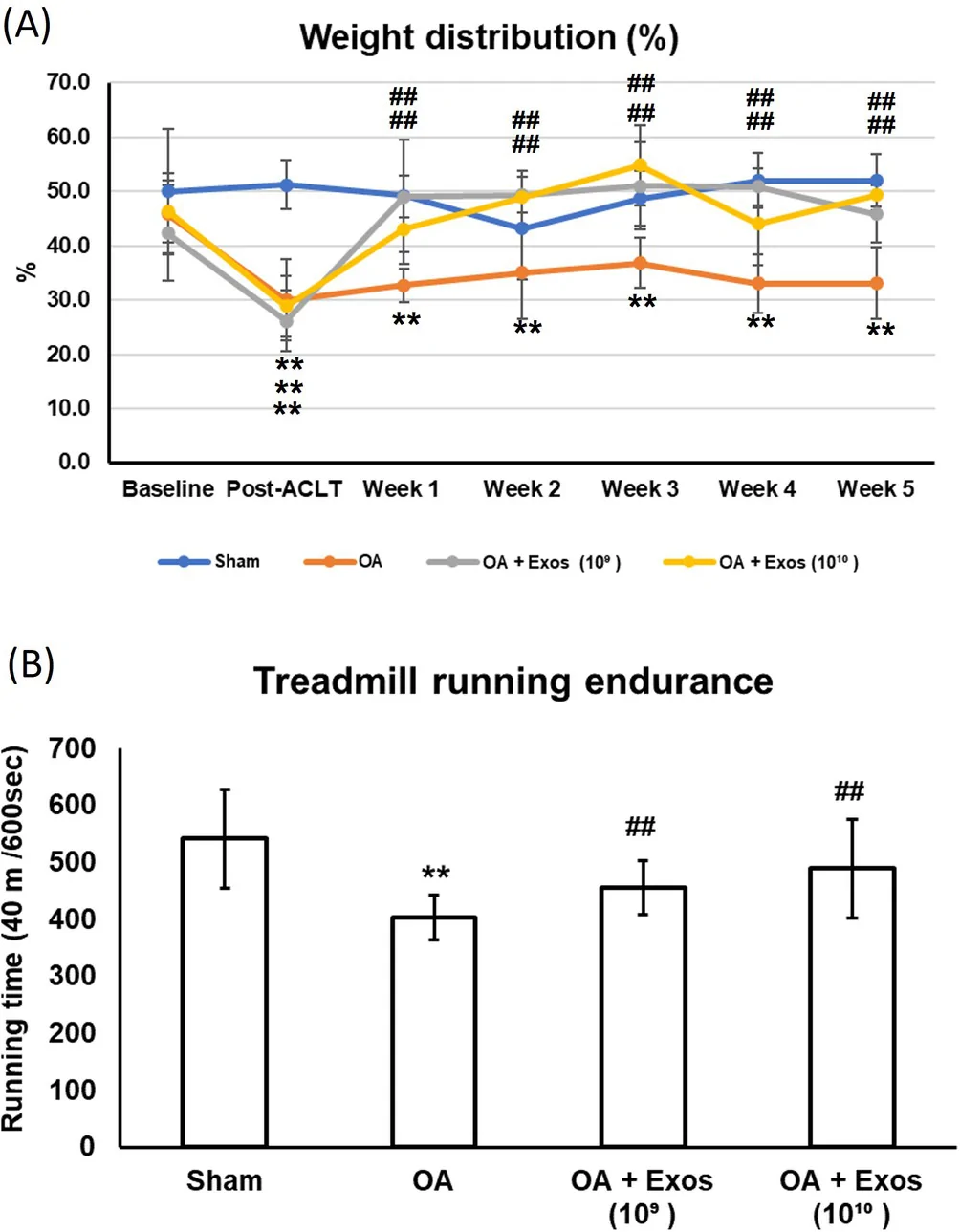
Human UCMSC‐derived exosomes improve functional recovery following anterior cruciate ligament transection (ACLT). (A) Weekly changes in hind limb weight‐bearing distribution after ACLT. Human UCMSC‐derived exosomes significantly improved weight‐bearing ratio throughout the treatment period compared with untreated osteoarthritis (OA) animals. (B) Treadmill running endurance measured 6 weeks after surgery. Both exosome‐treated groups exhibited significantly greater running endurance than the OA group, with no significant difference between the two doses. Data are presented as the mean ± SEM. Statistical analysis was performed using one‐way ANOVA followed by Scheffé's post hoc test. **p < 0.01 versus Sham; ^##^ *p* < 0.01 versus OA.

### 3.4 hUCMSC‐Exos Attenuate Cartilage Degeneration in ACLT‐Induced OA

Macroscopic examination revealed severe cartilage erosion, surface fibrillation, and osteophyte formation in untreated OA joints, particularly over the medial tibial plateau (Figure 6A). In contrast, joints treated with hUCMSC‐Exos displayed smoother articular surfaces with substantially reduced cartilage erosion. These morphological improvements were reflected by significantly lower macroscopic cartilage degeneration scores compared with the untreated OA group (Figure 6B).

**FIGURE 6:**
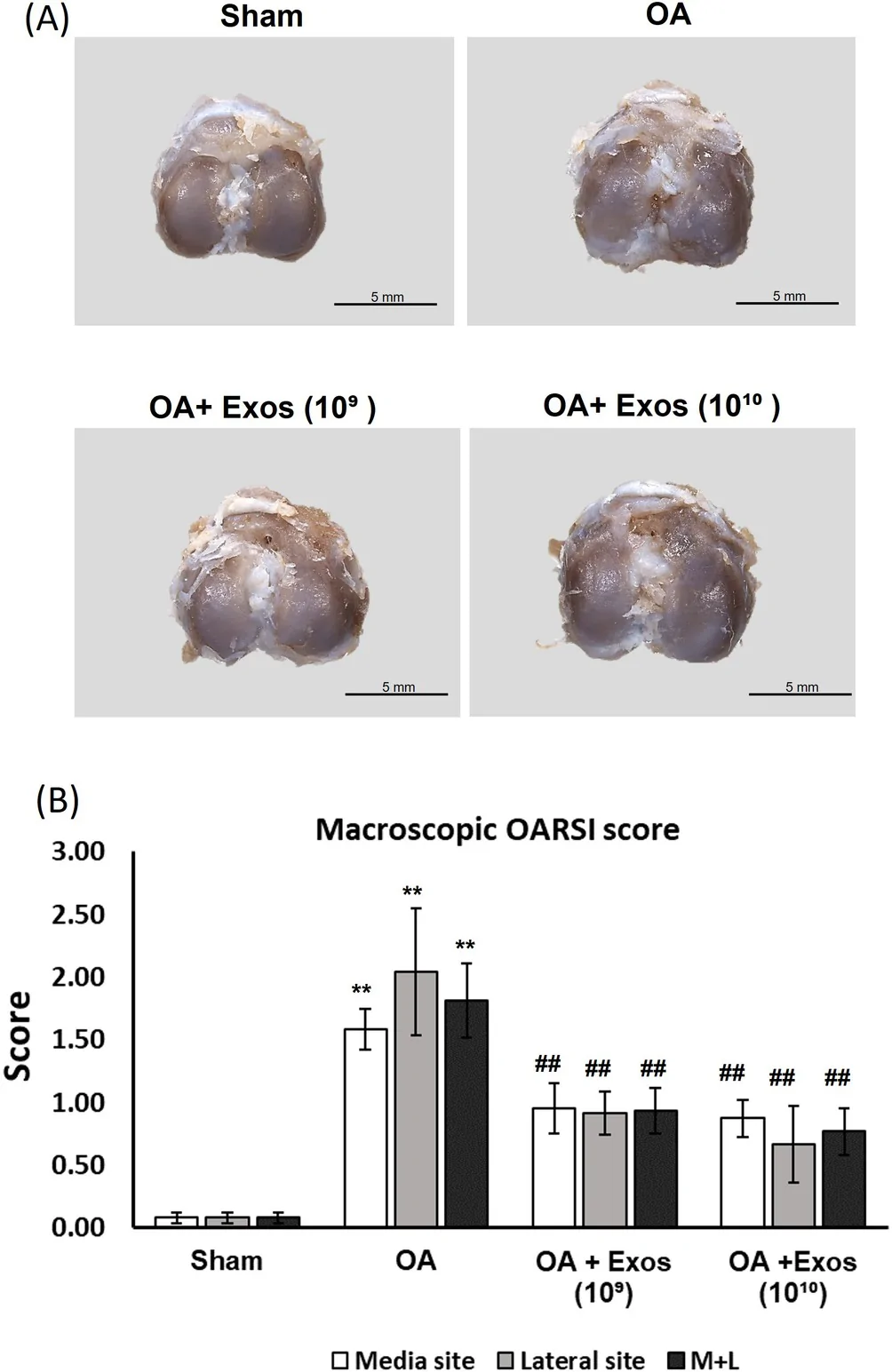
Human UCMSC‐derived exosomes attenuate macroscopic cartilage degeneration in ACLT‐induced OA. (A) Representative gross appearance of the tibial plateau from the Sham, OA, OA + hUCMSC‐derived exosomes (10^9^ particles), and OA + hUCMSC‐derived exosomes (10^10^ particles) groups at the end of the experimental period. Scale bar = 5 mm. (B) Macroscopic cartilage degeneration scores of the medial and lateral tibial plateau. Cartilage damage was graded according to the Osteoarthritis Research Society International (OARSI) scoring system. Data are presented as the mean ± SEM. Statistical analysis was performed using one‐way ANOVA followed by Scheffé's post hoc test. **p < 0.01 versus Sham; ^##^ *p* < 0.01 versus OA.

Histological evaluation using Safranin O/Fast Green staining demonstrated marked loss of proteoglycan staining and structural cartilage degeneration in OA joints, whereas hUCMSC‐Exos treatment preserved cartilage architecture and Safranin O‐positive matrix staining (Figure 7A). Consistently, histological OARSI scores were significantly reduced in both treatment groups (Figure 7B). Quantitative image analysis further demonstrated restoration of cartilage GAG deposition following exosome treatment, with the 10^10^‐particle group showing the greatest increase (Figure 7C).

**FIGURE 7:**
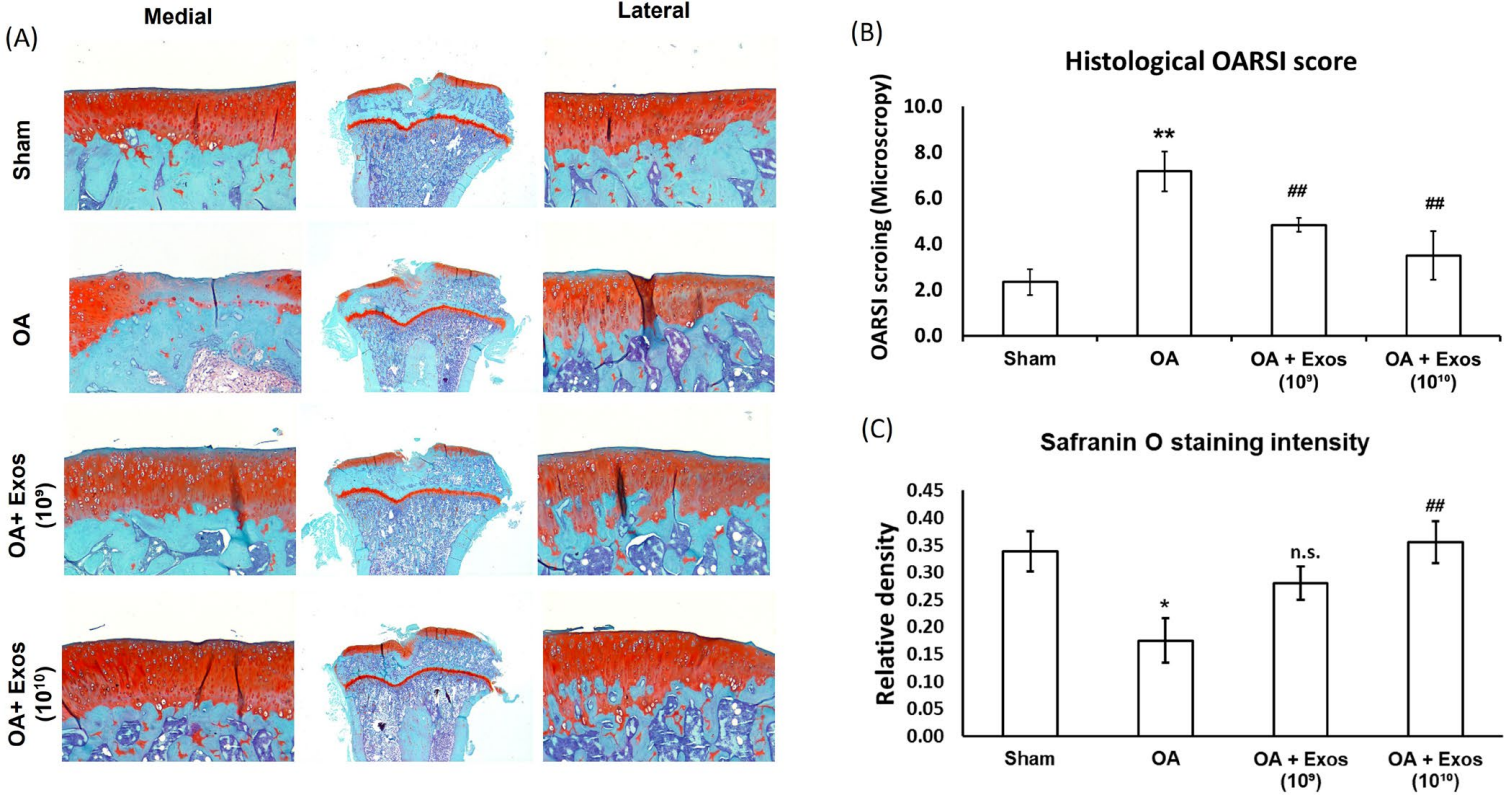
Human UCMSC‐derived exosomes preserve articular cartilage structure in the ACLT‐induced OA model. (A) Representative Safranin O/Fast Green‐stained sections of knee joints from the Sham, OA, OA + UCMSC‐derived exosomes (10^9^ particles), and OA + UCMSC‐derived exosomes (10^10^ particles) groups. (B) Histological cartilage degeneration was evaluated using the OARSI scoring system. (C) Quantitative analysis of Safranin O staining intensity was performed to assess proteoglycan preservation in the articular cartilage. Data are presented as the mean ± SEM. Statistical analysis was performed using one‐way ANOVA followed by Scheffé's post hoc test. *p < 0.05, **p < 0.01 versus Sham; ^#^ *p* < 0.05, ^##^ *p* < 0.01 versus OA.

Immunohistochemical analysis further supported these observations. Compared with the sham group, OA cartilage exhibited marked loss of Type II collagen accompanied by increased MMP13 expression. Treatment with hUCMSC‐Exos significantly restored Type II collagen expression while reducing MMP13 immunoreactivity (Figure 8). The increase in Type II collagen was most pronounced in the 10^10^‐particle group, whereas both treatment groups showed comparable suppression of MMP13. Collectively, these findings demonstrate that hUCMSC‐Exos preserve cartilage extracellular matrix integrity while attenuating cartilage catabolism in experimental OA.

**FIGURE 8:**
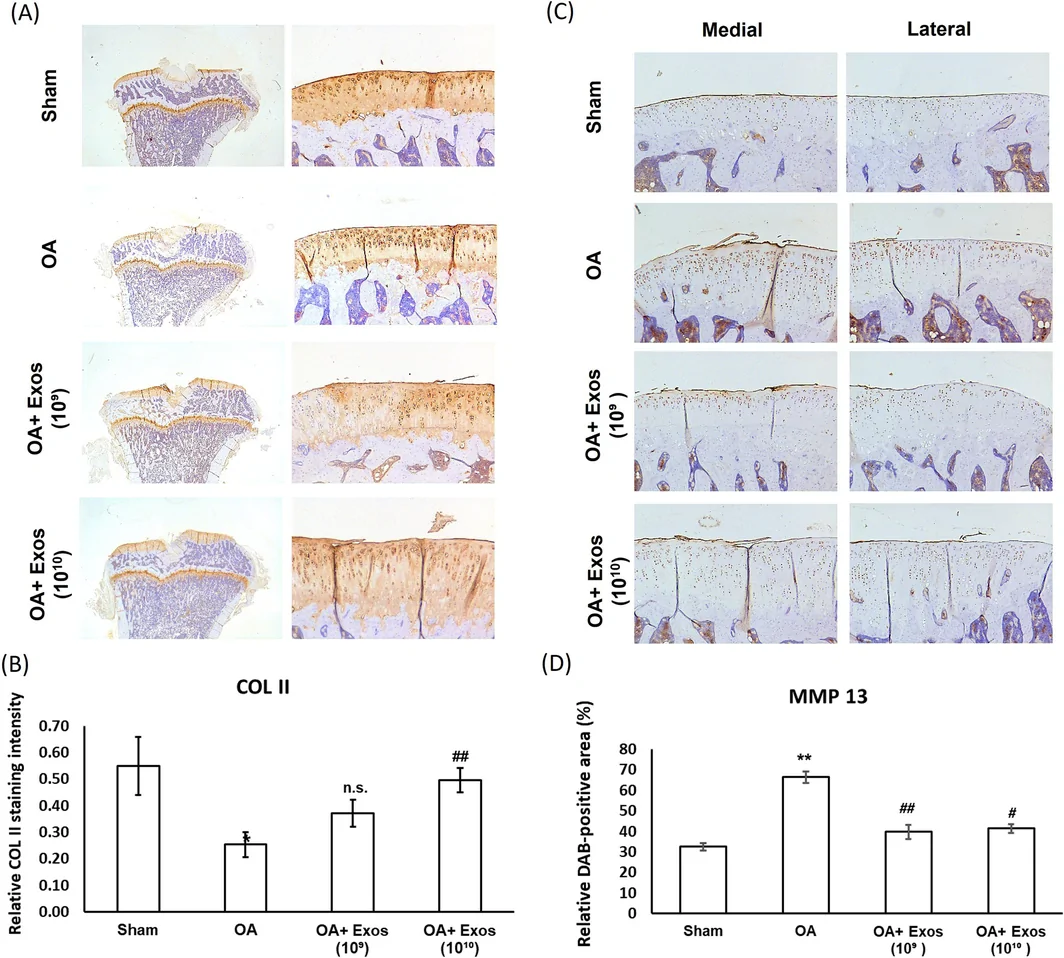
Human UCMSC‐derived exosomes preserve Type II collagen expression and suppress MMP13 expression in articular cartilage of ACLT‐induced OA model. (A) Representative immunohistochemical staining for Type II collagen (COL II) in the articular cartilage of the Sham, OA, OA + hUCMSC‐derived exosomes (10^9^ particles), and OA + hUCMSC‐derived exosomes (10^10^ particles) groups. (B) Quantitative analysis of COL II immunostaining. (C) Representative immunohistochemical staining for MMP‐13 in the medial and lateral tibial plateau. (D) Quantitative analysis of MMP‐13 immunostaining. Data are presented as the mean ± SEM. Statistical analysis was performed using one‐way ANOVA followed by Scheffé's post hoc test. *p < 0.05, **p < 0.01 versus Sham; ^#^ *p* < 0.05, ^##^ *p* < 0.01 versus OA.

## 4 Discussion

The present study demonstrates that repeated intra‐articular administration of hUCMSC‐Exos effectively attenuated OA progression by preserving cartilage extracellular matrix homeostasis and, importantly, restoring joint function. hUCMSC‐Exos suppressed inflammatory and catabolic responses, enhanced anabolic matrix synthesis, reduced cartilage degeneration, and improved weight‐bearing behavior and treadmill endurance. These findings indicate that structural preservation of articular cartilage translates into measurable functional recovery, thereby strengthening the translational potential of hUCMSC‐Exos as a cell‐free therapeutic strategy for OA.

Increasing evidence suggests that the therapeutic benefits of MSCs are mediated predominantly through paracrine signaling rather than durable engraftment [3]. Among the available MSC sources, hUCMSCs have attracted particular interest because of their high proliferative capacity, low immunogenicity, and non‐invasive procurement. Consistent with these advantages, a recent systematic review by Sourugeon et al. reported that 92.6% (25/27) of preclinical studies evaluating UCMSC‐based therapies demonstrated significant improvements in macroscopic, histological, or immunohistochemical outcomes, with consistent therapeutic benefits observed at both the cartilage and synovial levels [15]. Early clinical studies have likewise shown that intra‐articular administration of hUCMSCs is safe and associated with improvements in pain and knee function [16]. Previous studies have demonstrated that hUCMSC‐Exos suppress inflammatory responses, attenuate chondrocyte apoptosis, restore Type II collagen expression, reduce MMP13 expression, and preserve cartilage structure in experimental OA models [17, 18]. However, these studies have evaluated therapeutic efficacy primarily using molecular and histological outcomes. In contrast, our study demonstrates that the structural benefits of hUCMSC‐Exos are accompanied by significant improvements in joint function, thereby strengthening the translational relevance of this therapeutic strategy.

In the present study, hUCMSC‐Exos attenuated the IL‐1β‐induced inflammatory and catabolic response in chondrocytes, as evidenced by reduced IL1B and MMP13 expression, while restoring cartilage matrix‐related markers, including TIMP1, ACAN, and COL2A1. These findings are consistent with previous studies demonstrating anti‐inflammatory and cartilage‐protective effects of hUCMSC‐Exos in experimental OA [17, 18]. A recent study further identified the circ_0006646/IGF2BP2/CDH11 axis as a regulator of IL‐1β‐induced chondrocyte injury and extracellular matrix degradation, highlighting the molecular complexity of OA‐associated cartilage catabolism [19]. Importantly, these molecular effects were accompanied by increased sGAG production in vitro and preservation of Type II collagen in vivo, suggesting that the observed modulation of inflammatory and catabolic activity was associated with maintenance of cartilage matrix homeostasis. In the ACLT model, cartilage degeneration and matrix‐catabolic activity begin early after joint destabilization and progress over time [20, 21]. Our findings therefore suggest that hUCMSC‐Exos may intervene during this progressive catabolic process by preserving the balance between cartilage matrix degradation and synthesis. Nevertheless, because inflammatory responses were evaluated primarily in chondrocytes, the present study does not establish whether hUCMSC‐Exos exert broader anti‐inflammatory or immunomodulatory effects within the joint.

Importantly, functional recovery represents a key translational outcome because improvement in joint use and physical performance is ultimately more clinically relevant than structural cartilage preservation alone. In the present study, hUCMSC‐Exos improved both hind limb weight‐bearing and treadmill endurance, which reflect complementary aspects of joint function. Weight‐bearing asymmetry reflects preferential unloading of the affected limb and is commonly used as a pain‐related behavioral measure in experimental OA, whereas treadmill endurance provides a complementary assessment of sustained locomotor performance and exercise tolerance [12, 22]. The improvement in both outcomes suggests that the therapeutic effects of hUCMSC‐Exos were not limited to histological cartilage preservation but translated into meaningful recovery of joint function. Notably, weight‐bearing improved relatively early during treatment, suggesting that reduced joint discomfort or modulation of the local inflammatory environment may precede detectable structural improvement. In contrast, improved treadmill endurance may reflect the cumulative effects of better joint loading tolerance and preservation of cartilage integrity over time. These distinct but complementary functional responses strengthen the translational relevance of the observed structural and molecular benefits of hUCMSC‐Exos.

Although both doses demonstrated therapeutic efficacy, no clear dose‐dependent response was observed. The higher dose yielded greater improvements in selected cartilage matrix‐related outcomes; however, functional recovery and MMP13 suppression were comparable between the two groups. Nonlinear dose–response relationships have also been reported for MSC‐derived exosomes in other experimental models, with an intermediate dose producing greater therapeutic benefits than either lower or higher doses [23]. In addition, a recent dose‐escalation study of intra‐articular hUCMSC‐Exos in patients with knee OA found no significant differences among dose groups at the same follow‐up time point [24]. These findings suggest that increasing the exosome dose does not necessarily result in a proportional enhancement of biological efficacy. The apparent plateau may reflect saturation of cellular uptake or downstream signaling, although these mechanisms were not directly examined in the present study. In addition, structural, molecular, and functional outcomes may have distinct dose–response thresholds. Therefore, our findings support the existence of an effective therapeutic range rather than a simple linear dose–response relationship. Further dose‐ranging studies incorporating pharmacokinetic analyses and validated potency assays are warranted to determine the minimum effective and optimal doses of hUCMSC‐Exos.

Several limitations should be acknowledged. First, the molecular mechanisms underlying the therapeutic effects of hUCMSC‐Exos were inferred from cartilage‐related molecular and histological changes, and the specific active cargo and signaling pathways were not directly investigated. Second, the study focused primarily on articular cartilage and functional outcomes; other whole‐joint changes, including synovial inflammation and subchondral bone remodeling, were not systematically evaluated. Finally, pharmacokinetics, biodistribution, long‐term safety, and durability after treatment cessation were not assessed. Further translation will therefore require standardized exosome manufacturing and characterization, optimization of dosing, and validation of efficacy and safety in larger animal models.

In conclusion, hUCMSC‐Exos preserved cartilage extracellular matrix homeostasis, attenuated structural degeneration, and restored joint function in experimental OA. By demonstrating that structural preservation is accompanied by meaningful functional recovery, the present study extends current evidence supporting exosome‐based therapy and provides a strong preclinical rationale for the future clinical development of disease‐modifying therapies for OA.

## Funding

The authors acknowledge the grant support for this study provided by the National Science and Technology Council of Taiwan (grant numbers: NSTC 113‐2314‐B‐037‐084‐MY3, 113‐2314‐B‐037‐008, 112‐2314‐B‐037‐097‐MY3, NSTC‐113‐2314‐B‐037‐086‐MY3, 114‐2926‐I‐037‐501–G, NSTC‐114‐2314‐B‐037‐050‐MY3), the grant from National Health Research Institute of Taiwan (grant numbers: NHRI‐EX114‐11224EI), Kaohsiung Medical University Orthopaedic Research Center (ORC) and Kaohsiung Medical University of Regenerative Medicine, Cell Therapy Research Center (RCC) (grant number: KMU‐TC115A02), Kaohsiung Medical University (grant number: NSYSU‐KMU‐115‐P21), Kaohsiung Medical University Hospital (grant numbers: KMUH‐SI11515, KMUH‐SH11504, KMUH‐114‐4R54, KMUH‐DK(N)115001), Kaohsiung Medical University Gangshan Hospital (KMUGH‐113‐R002).

## Conflicts of Interest

ExoOne Bio Co. Ltd. provided the UCMSC‐Exos used in this study. The authors declare no conflicts of interest.

## Supporting information


**Data S1:** Supporting Information.

## Data Availability

The data that support the findings of this study are available from the corresponding author upon reasonable request.

## References

[1] GBD 2021 Osteoarthritis Collaborators , “Global, Regional, and National Burden of Osteoarthritis, 1990–2020 and Projections to 2050: A Systematic Analysis for the Global Burden of Disease Study 2021,” Lancet Rheumatology 5, no. 9 (2023): e508–e522. 10.1016/S2665-9913(23)00163-7 PMC10477960 37675071

[2] Y. Ouyang and M. Dai , “Global, Regional, and National Burden of Knee Osteoarthritis: Findings From the Global Burden of Disease Study 2021 and Projections to 2045,” Journal of Orthopaedic Surgery and Research 20, no. 1 (2025): 766. 10.1186/s13018-025-06140-0 PMC12351899 40804406

[3] X. Han , R. Liao , X. Li , et al., “Mesenchymal Stem Cells in Treating Human Diseases: Molecular Mechanisms and Clinical Studies,” Signal Transduction and Targeted Therapy 10, no. 1 (2025): 262. 10.1038/s41392-025-02313-9 PMC12371117 40841367

[4] H. Kong , J. Zheng , J. Han , et al., “The Crosstalk Among Macrophages, Chondrocytes and Mesenchymal Stem Cells in Osteoarthritis: The Role of Extracellular Vesicles,” Stem Cell Research & Therapy 16, no. 1 (2025): 553. 10.1186/s13287-025-04659-x PMC12512754 41074166

[5] F. Aziziyan , S. S. Asl , M. Mahdipour , R. N. Fard , and M. Sheykhhasan , “Mesenchymal Stem Cell‐Derived Extracellular Vesicles in Musculoskeletal Regeneration: Mechanisms, Applications, and Future Prospects,” Stem Cell Research & Therapy 17, no. 1 (2026): 66. 10.1186/s13287-025-04879-1 PMC12866515 41484803

[6] R. C. Lai , R. W. Yeo , and S. K. Lim , “Mesenchymal Stem Cell Exosomes,” Seminars in Cell & Developmental Biology 40 (2015): 82–88. 10.1016/j.semcdb.2015.03.001 25765629

[7] Y. Yaghoubi , A. Movassaghpour , M. Zamani , M. Talebi , A. Mehdizadeh , and M. Yousefi , “Human Umbilical Cord Mesenchymal Stem Cells Derived‐Exosomes in Diseases Treatment,” Life Sciences 233 (2019): 116733. 10.1016/j.lfs.2019.116733 31394127

[8] H. Yang , Y. Zhou , B. Ying , X. Dong , Q. Qian , and S. Gao , “Effects of Human Umbilical Cord Mesenchymal Stem Cell‐Derived Exosomes in the Rat Osteoarthritis Models,” Stem Cells Translational Medicine 13, no. 8 (2024): 803–811. 10.1093/stcltm/szae031 PMC11328936 38913985

[9] S. Tang , P. Chen , H. Zhang , et al., “Comparison of Curative Effect of Human Umbilical Cord‐Derived Mesenchymal Stem Cells and Their Small Extracellular Vesicles in Treating Osteoarthritis,” International Journal of Nanomedicine 16 (2021): 8185–8202. 10.2147/IJN.S336062 PMC8687685 34938076

[10] D. Bachurski , M. Schuldner , P. H. Nguyen , et al., “Extracellular Vesicle Measurements With Nanoparticle Tracking Analysis—An Accuracy and Repeatability Comparison Between NanoSight NS300 and ZetaView,” Journal of Extracellular Vesicles 8, no. 1 (2019): 1596016. 10.1080/20013078.2019.1596016 PMC6450530 30988894

[11] A. H. Cory , T. C. Owen , J. A. Barltrop , and J. G. Cory , “Use of an Aqueous Soluble Tetrazolium/Formazan Assay for Cell Growth Assays in Culture,” Cancer Communications 3, no. 7 (1991): 207–212. 10.3727/095535491820873191 1867954

[12] C. H. Chen , M. L. Ho , L. H. Chang , et al., “Parathyroid Hormone‐(1‐34) Ameliorated Knee Osteoarthritis in Rats via Autophagy,” Journal of Applied Physiology (Bethesda, MD: 1985) 124, no. 5 (2018): 1177–1185. 10.1152/japplphysiol.00871.2017 29357491

[13] L. H. Chang , S. C. Wu , C. H. Chen , et al., “Exosomes Derived From Hypoxia‐Cultured Human Adipose Stem Cells Alleviate Articular Chondrocyte Inflammaging and Post‐Traumatic Osteoarthritis Progression,” International Journal of Molecular Sciences 24, no. 17 (2023): 13414. 10.3390/ijms241713414 PMC10487932 37686220

[14] H. T. Huang , T. L. Cheng , C. J. Ho , et al., “Intra‐Articular Injection of (−)‐Epigallocatechin 3‐Gallate to Attenuate Articular Cartilage Degeneration by Enhancing Autophagy in a Post‐Traumatic Osteoarthritis Rat Model,” Antioxidants (Basel) 10, no. 1 (2020): 8. 10.3390/antiox10010008 PMC7824012 33374730

[15] Y. Sourugeon , A. Boffa , C. Perucca Orfei , et al., “Cell‐Based Therapies Have Disease‐Modifying Effects on Osteoarthritis in Animal Models: A Systematic Review by the ESSKA Orthobiologic Initiative. Part 3: Umbilical Cord, Placenta, and Other Sources for Cell‐Based Injectable Therapies,” Knee Surgery, Sports Traumatology, Arthroscopy 33, no. 5 (2025): 1695–1708. 10.1002/ksa.12472 PMC12022835 39302089

[16] Z. Xiao , X. Wang , C. Li , L. Luo , and W. Li , “Effects of the Umbilical Cord Mesenchymal Stem Cells in the Treatment of Knee Osteoarthritis: A Systematic Review and Meta‐Analysis,” Medicine (Baltimore) 103, no. 46 (2024): e40490. 10.1097/MD.0000000000040490 PMC11575993 39560593

[17] S. Wang , W. Jiang , S. Lv , et al., “Human Umbilical Cord Mesenchymal Stem Cells‐Derived Exosomes Exert Anti‐Inflammatory Effects on Osteoarthritis Chondrocytes,” Aging (Albany NY) 15, no. 18 (2023): 9544–9560. 10.18632/aging.205034 PMC10564422 37724890

[18] S. Qin , A. Zhang , L. Duan , F. Lin , and M. Zhao , “Exosomes Extracted From Human Umbilical Cord MSCs Contribute to Osteoarthritic Cartilage and Chondrocytes Repair Through Enhancing Autophagy While Suppressing the Wnt/β‐Catenin Pathway,” Tissue Engineering and Regenerative Medicine 22, no. 5 (2025): 719–733. 10.1007/s13770-025-00716-x PMC12209105 40232653

[19] M. Y. Hua , G. L. Wang , W. H. Duan , and X. H. Tang , “Circ_0006646 Promotes the Progression of Osteoarthritis via Upregulating CDH11 Expression in an IGF2BP2‐Dependent Manner,” Kaohsiung Journal of Medical Sciences 41, no. 8 (2025): e70031. 10.1002/kjm2.70031 PMC12407339 40387410

[20] C. Huang , G. Zhang , Y. K. Ma , X. N. Ma , and S. C. Lv , “Melatonin Exerts Chondroprotective Effects Against Osteoarthritis by Promoting PI3K/AKT/FoxO3‐Mediated Mitophagy,” Kaohsiung Journal of Medical Sciences 42, no. 5 (2026): e70131. 10.1002/kjm2.70131 PMC13182593 41190713

[21] M. Pickarski , T. Hayami , Y. Zhuo , and L. T. Duong , “Molecular Changes in Articular Cartilage and Subchondral Bone in the Rat Anterior Cruciate Ligament Transection and Meniscectomized Models of Osteoarthritis,” BMC Musculoskeletal Disorders 12 (2011): 197. 10.1186/1471-2474-12-197 PMC3176489 21864409

[22] C. B. Hamilton , M. A. Pest , V. Pitelka , A. Ratneswaran , F. Beier , and B. M. Chesworth , “Weight‐Bearing Asymmetry and Vertical Activity Differences in a Rat Model of Post‐Traumatic Knee Osteoarthritis,” Osteoarthritis and Cartilage 23, no. 7 (2015): 1178–1185. 10.1016/j.joca.2015.03.001 25771150

[23] Y. Zhang , Y. Zhang , M. Chopp , Z. G. Zhang , A. Mahmood , and Y. Xiong , “Mesenchymal Stem Cell‐Derived Exosomes Improve Functional Recovery in Rats After Traumatic Brain Injury: A Dose‐Response and Therapeutic Window Study,” Neurorehabilitation and Neural Repair 34, no. 7 (2020): 616–626. 10.1177/1545968320926164 PMC7329589 32462980

[24] Y. Wang , Y. Kong , J. Du , et al., “Injection of Human Umbilical Cord Mesenchymal Stem Cells Exosomes for the Treatment of Knee Osteoarthritis: From Preclinical to Clinical Research,” Journal of Translational Medicine 23 (2025): 641. 10.1186/s12967-025-06623-y PMC12153132 40500748

