# Supplementary material for: Human Umbilical Cord Mesenchymal Stem Cell‐Derived Exosomes Preserve Cartilage Homeostasis and Restore Joint Function in Experimental Osteoarthritis
Source: Kaohsiung J Med Sci. 2026 Nov 5:e70312. Online ahead of print. doi: 10.1002/kjm2.70312 (PMC13735434; doi:10.1002/kjm2.70312)
Supplement: Supplementary file 1 [file KJM2-9999-e70312-s001.docx]

Human IL-1β Forward:5’-GCA ATG AGG ATG ACT TGT TCT-3’;

Human IL-1β Reverse: 5’-GGT CAT TCT CCT GGA AGG TCT-3’ GTGGGC;

Human MMP13 Forward:5’-CTT CCC AAC CGT ATT GAT GCT-3’

Human MMP13 Reverse: 5’-CTG GTT TCC TGA GAA CAG GAG-3’

Human type X collagen Forward: 5’-GGTCTCGATGGTCCTAAGGG-3’

Human type X collagen5’ Reverse: 5’-GGATCCCCTTTAGACCCAGG-3’

Human TIMP-1 Forward:5’-CCCAGAGAGACACCAGAGAAC-3’

Human TIMP-1 Reverse: 5’-CACGA CTT GGC CCT GAT GAC-3’

Human type II collagen Forward: 5’GGGAGTAATGCAAGGACCAA

Human type II collagen Reverse: 5’-ATCATCACCAGGCTTTCCAG-3’

Human Aggrecan Forward: 5’-ACA GCT GGG GAC ATT AGT GG-3’

Human Aggrecan Reverse: 5-’GTG GAA TGC AGA GGT GGT TT-3’

Human GAPDH Forward: 5’-TCT CCT CTG ACT TCA ACA GCG AC-3’

Human GAPDH Reverse: 5’-CCC TGT TGC TGT AGC CAA ATT C-3’
